# Unveiling Human Proteome Signatures of Heart Failure with Preserved Ejection Fraction

**DOI:** 10.3390/biomedicines10112943

**Published:** 2022-11-16

**Authors:** Maria J. Sebastião, Henrique V. Almeida, Margarida Serra, Nazha Hamdani, Francisca Saraiva, André P. Lourenço, António S. Barros, Francisco Vasques-Nóvoa, Adelino Leite-Moreira, Paula M. Alves, Inês Falcão-Pires, Patrícia Gomes-Alves

**Affiliations:** 1 iBET, Instituto de Biologia Experimental e Tecnológica, Apartado 12, 2780-901 Oeiras, Portugal; 2 ITQB-NOVA, Instituto de Tecnologia Química e Biológica António Xavier, Universidade Nova de Lisboa, Av. Da República, 2780-157 Oeiras, Portugal; 3 Institut für Forschung und Lehre (IFL), Molecular and Experimental Cardiology, Ruhr University Bochum, 44791 Bochum, Germany; 4 Department of Cardiology, St. Josef-Hospital, Ruhr University Bochum, 44791 Bochum, Germany; 5 Institute of Physiology, Ruhr University Bochum, 44801 Bochum, Germany; 6 Department of Surgery and Physiology, Cardiovascular R&D Unit, Faculty of Medicine, University of Porto, 4200-319 Porto, Portugal; 7 Department of Internal Medicine, Centro Hospitalar Universitário de São João, 4200-319 Porto, Portugal; 8 Department of Cardiothoracic Surgery, Centro Hospitalar Universitário de São João, 4200-319 Porto, Portugal

**Keywords:** diabetes mellitus, heart failure with preserved ejection fraction, SWATH–MS proteomics, human cardiac tissue

## Abstract

Heart failure with preserved ejection fraction (HFpEF) is a highly prevalent but still poorly understood clinical entity. Its current pathophysiological understanding supports a critical role of comorbidities and their chronic effect on cardiac function and structure. Importantly, despite the replication of some HFpEF phenotypic features, to this day, experimental models have failed to bring new effective therapies to the clinical setting. Thus, the direct investigation of HFpEF human myocardial samples may unveil key, and possibly human-specific, pathophysiological mechanisms. This study employed quantitative proteomic analysis by advanced mass spectrometry (SWATH–MS) to investigate signaling pathways and pathophysiological mechanisms in HFpEF. Protein-expression profiles were analyzed in human left ventricular myocardial samples of HFpEF patients and compared with a mixed control group. Functional analysis revealed several proteins that correlate with HFpEF, including those associated with mitochondrial dysfunction, oxidative stress, and inflammation. Despite the known disease heterogeneity, proteomic profiles could indicate a reduced mitochondrial oxidative phosphorylation and fatty-acid oxidation capacity in HFpEF patients with diabetes. The proteomic characterization described in this work provides new insights. Furthermore, it fosters further questions related to HFpEF cellular pathophysiology, paving the way for additional studies focused on developing novel therapies and diagnosis strategies for HFpEF patients.

## 1. Introduction

Despite its high prevalence, surmounting health-costs, and increasing death toll, heart failure (HF) with preserved ejection fraction (HFpEF) remains one of the major unmet challenges in current healthcare. Whereas improvements have been made for HF with reduced ejection fraction (HFrEF), few advances have been made for patients with HFpEF, with just one pharmacological group (SGLT2i) having been recently shown to improve clinical outcomes [1]. The main culprit for the lack of effective therapeutic alternatives, according to most authors, is the high heterogeneity of the syndrome. Indeed, the key to success may be the identification of patient subgroups that may benefit from tailored therapeutic interventions.

The current consensus regarding the key pathophysiological aspects of HFpEF links comorbidity-driven chronic low-grade inflammation, endothelial dysfunction, alterations in fluid balance, and multiple organ dysfunctions to functional and structural changes (atrial fibrillation, ventricular hypertrophy, and fibrosis) that, overall, impair cardiac reserve despite preserved ejection fraction and gross systolic function indexes [2,3,4,5].

There is a clear need to understand the protein-mediated mechanisms of human HFpEF. Despite mimicking some HFpEF phenotypic features, to this day, HfpEF experimental models have failed to bring new effective therapies to the clinical setting. In this regard, the evaluation of human myocardial biopsies [6,7,8] and plasma protein profiles [2,9,10] from HFpEF patients is valuable and has suggested new putative therapeutic targets and supported an important role of the comorbidity-driven activation of inflammatory signaling pathways. Indeed, in previously reported studies, circulating inflammatory protein biomarkers appear to corroborate the comorbidity-inflammation paradigm for HFpEF [2,9,10,11,12,13,14]. However, these previous studies used targeted methodologies aimed at only a specific subset of cardiomyocyte features or plasma proteins. 

Herein, we aimed to characterize the proteome of human HFpEF left ventricle myocardial samples. These samples were thoroughly characterized by untargeted mass-spectrometry-based quantitative proteomic analysis (SWATH–MS) to investigate signaling pathways and mechanisms associated with HFpEF and linked to comorbidities. The knowledge generated here will contribute to unveiling human myocardial signatures of HFpEF and its correlation with known comorbidities, potentially improving experimental modeling, diagnosis, and therapeutics. 

## 2. Materials and Methods

### 2.1. Heart-Tissue Collection and Processing

All procedures were performed according to the Declaration of Helsinki and were approved by the local ethics committee and data-protection authority (CES2006, 35/2017, and 1774/2017). Myocardial samples were collected between 2010 and 2018 from (i) HFpEF patients, during LV myocardial biopsy during coronary artery bypass grafting surgery (n = 11) or due to non-confirmed suspicion of infiltrative or inflammatory cardiomyopathy (n = 5), and (ii) a mixed control group, composed of explanted donor hearts (n = 5) and aortic stenosis patients submitted to aortic valve replacement surgery (n = 7). Sample collection during aortic valve replacement was carried out at the interventricular septum, as part of the Morrow procedure. Sample collection during coronary artery bypass grafting was carried out during target exposition for distal graft anastomosis. Patients were diagnosed according to diagnostic guidelines contemporary to the date of procedures. The echocardiography protocol followed the 2016 ESC HF Guidelines recommendations [15] and looked for the presence of relevant structural heart disease (left ventricular hypertrophy and/or left atrial enlargement) and/or evidence of diastolic dysfunction. Patient characteristics and clinical data are depicted in Table 1. Individualized patient data can be accessed in Supplementary File S1.

Proteins were extracted using the Norgen kit (Norgen Biotek), following the manufacturer’s protocol. Briefly, frozen LV myocardium samples were homogenized in the presence of ceramic beads (ø = 2.8 mm) and kit lysis buffer (200 µL or 300 µL if sample ≥ 5 mg) using a PreCellys^®^ Homogenizer. Then, samples were transferred to a new tube and incubated at 55 °C for 10 min. Subsequently, precipitation and wash steps were performed to purify proteins. Extracted protein was quantified by Bradford protein assay kit (ThermoScientific). Samples were kept at −80 °C until further analysis.

Proteins were subjected to electrophoresis and digested with trypsin, as described previously [16,17]. 

### 2.2. Proteomics

#### 2.2.1. Generation of the Spectral Reference Library 

Each sample (2.5 μg) was used for information-dependent acquisition (IDA) analysis by NanoLC–MS using TripleTOF 6600 (ABSciex), as described previously [16,17]. The spectral library was created by combining all IDA raw files using ProteinPilot software (v5.0 ABSciex) 

#### 2.2.2. SWATH–MS Analysis and Targeted Data Extraction

For quantitative analysis, 2.5 μg of each sample was subjected to 3 SWATH runs, as previously described [16,17]. A total of 1043 proteins were quantified under these conditions. The full list of quantified proteins can be accessed in Supplementary File S2. All proteomic raw data have been deposited in the ProteomeXchange Consortium (http://proteomecentral.proteomexchange.org, accessed on 12 May 2022) via the PRIDE partner repository with the dataset identifier PXD033876.

#### 2.2.3. Proteomic Data Analysis

SWATH log2 normalized data from 3 different MS runs were adjusted for batch effects with the parametric empirical Bayes ComBAT framework, using surrogate variable analysis (sva) R package version 3.42.0 [18]. Normalized and batch-corrected proteomic data can be accessed in Supplementary File S3.

To identify differentially expressed proteins, quantitative data from SWATH was analyzed using student’s *t*-test using GraphPad Prism6 (GraphPad Software Inc., San Diego, CA, USA). The resulting *p*-values and fold changes indicated up- and downregulated proteins. Differentially expressed proteins were defined as those that showed a fold-change greater than 1.5 (upregulated) or lower than 0.67 (downregulated) and *p*-values lower than 0.05. Principal component analysis and hierarchical clustering heatmap analysis were performed using Perseus software environment [19]. 

Pathway analysis was performed using Ingenuity Pathway Analysis software (IPA, Quiagen, Germany) by uploading the quantified protein list (including only proteins with *p*-value ≤ 0.05) and the respective fold change. A statistically significant representation of biological functions and canonical pathways was identified based on the IPA *p*-value, displayed as −log (*p*-value). The IPA *p*-value is a probability score calculated by considering the total number of proteins known to be associated with a given function or pathway, and their representation in the experimental dataset. The prediction of inhibition and the activation of biological functions and canonical pathways was based on the IPA z-score, a statistical measure of the match between the expected relationship direction and the observed protein expression, resulting in the activation (z ≥ 2) or inhibition (z ≤ −2) of the respective pathway. 

Statistical analyses were performed with GraphPad Prism6 (GraphPad Software Inc., San Diego, CA, USA). Data were analyzed by One Way ANOVA Tukey test or Student’s *t*-test. *p*-values below 0.05 were considered significant.

## 3. Results and Discussion

Proteomic analysis of 27 human ventricular myocardium samples, including 16 samples from patients with HFpEF and 11 samples from patients without HFpEF, resulted in the identification and quantification of a total of 1043 proteins. In addition, relative abundances were further normalized between analytical batches, leaving a total of 1015 analysis-ready proteins. A full list of raw and normalized protein quantification data can be accessed in Supplementary Files S2 and S3 and ProteomeXchange Consortium via the PRIDE partner repository with the dataset identifier PXD033876 (http://proteomecentral.proteomexchange.org, accessed on 12 May 2022).

**Figure 1:**
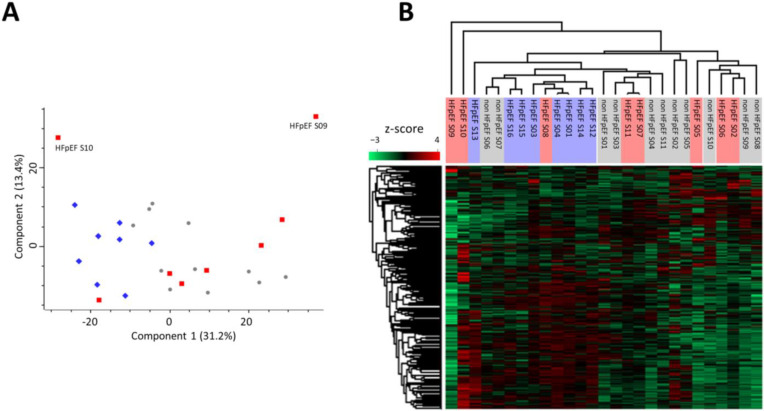
Heterogeneity of samples shown by dispersion of protein-expression profiles. (**A**) Principal component analysis (PCA) of all samples (PCA settings: unsupervised; weighting—none; scaling—Pareto). Scores for PC1 (31.2%) versus PC2 (13.4%) are displayed. (Blue diamonds: HFpEF samples; red squares: HFpEF + DM samples; and grey circles: non HFpEF samples). (**B**) Hierarchical clustering (Pearson correlation) heatmap of normalized protein quantifications (z-score, n = 1015).

Principal component analysis (PCA) and hierarchical clustering revealed high heterogeneity between samples, with no distinct clustering between HFpEF and non-HFpEF groups, with two HFpEF samples (HFpEF S09 and HFpEF S10) presenting a more distinct proteomic profile (Figure 1). Such divergent profiles could not be explained by the patient demographic, analytical, and echocardiographic data available for these samples (Table 1).

When investigating the data of those two samples more deeply, we could observe higher levels of myosins (MYL1, MYL6B) and troponins (TNNT1, TNNT3, TNNC2, and TNNI2) in HFpEF S09 (Supplementary File S2), suggesting a higher proportion of cardiomyocytes in this biopsy sample. In addition, sample HFpEF S10 revealed a protein expression profile indicative of inflammation, with higher levels of neutrophil serine proteases CTSG, ELANE, and PRTN3, as well as increased MMP9 and several other proteins associated with an inflammatory response such as AZU1, BPI, and C4A (Supplementary File S2). Although both samples presented these apparent differences at the proteomic profile level, they were included in all subsequent analyses as they illustrate human biopsy samples’ inherent donor-to-donor harvesting variability and HFpEF intrinsic heterogeneity.

### 3.1. Proteomic Signature of Myocardium Tissue from HFpEF Patients

By aiming to obtain a better molecular characterization of HFpEF myocardium, we analyzed and compared the protein-expression profiles of HFpEF samples vs. non-HFpEF samples. Of the 1015 identified proteins, SAA1 protein presented lower expression levels (FC ≤ 0.67 and *p*-value ≤ 0.05), 9 proteins presented significantly higher expression levels (FC ≥ 1.5 and *p*-value ≤ 0.05), and COMT and EHD1 proteins also presented a tendency for higher expression levels (*p*-value ≤ 0.05) when comparing both sample groups (Table 2 and Supplementary File S4).

Unlike other HF conditions (such as HFrEF), in which there is a triggering event such as acute myocardial infarction, HFpEF pathophysiology mechanisms are believed to be mainly caused by inflammation arising from cardiovascular comorbidities [2,5,20]. Such a systemic pro-inflammatory environment leads to endothelial dysfunction, oxidative stress, mitochondrial dysfunction, cardiomyocyte hypertrophy, calcium cycling impairment, and extracellular matrix deposition, leading to myocardial fibrosis, hypertrophy, and failure [20,21,22].

The upregulation of peroxiredoxin-like 2A (PRXL2A, also known as PAMM, or FAM213A, Table 2) points to the negative regulation of inflammation in the myocardium of HFpEF patients. In addition, PRXL2A has been reported to act as a negative regulator of macrophage-induced inflammation [23] and is also involved in protection against cellular oxidative stress [24,25].

The myocardium is especially susceptible to oxidative stress and mitochondrial respiration dysfunction due to its metabolic dependence on aerobic respiration [26]. Cardiac dysfunctions, including HFpEF, are frequently associated with deficits in the mitochondrial respiratory chain, namely, with NADH:ubiquinone oxidoreductase (complex I) decreased activity [27,28,29]. NDUFA11, a subunit of complex I, which plays a a role in complex assembly and stabilization, was found to be upregulated in HFpEF samples, which might indicate the upregulation of NDUFA11 as a mechanism to compensate for complex I dysregulation. Another mitochondrial protein found to be increased in HFpEF vs. non-HFpEF samples was the mitochondrial apoptosis-inducing factor 1 (AIFM, also designated as PCDC8). This protein is released from the mitochondria to the cytosol and nucleus upon apoptotic stimuli. Besides acting as a caspase-independent proapoptotic factor, several reports have attributed another seemingly paradoxical role of AIFM1 in protection against oxidative stress via the metabolism of reactive oxygen species (ROS) [30,31], which is especially relevant in hypertrophic cardiomyocytes [32].

The laminin subunit alpha 4 (LAMA4) was also identified as being upregulated in HFpEF samples. LAMA4 is part of laminin 8 and 9, which are major constituents of heart-basement membranes [33]. Increasing hemodynamic loads in hypertensive patients are sensed by cardiomyocytes, fibroblasts, and endothelial cells, leading to alterations in the basement membrane laminin composition, followed by cardiac hypertrophy. Mice deficient in LAMA4 were reported to present augmented cardiac hypertrophy and increased levels of HF [34].

Other proteins found to be upregulated in HFpEF include PRKACA (involved in contraction of cardiac muscle via phosphorylation of RyR2 channel) [35]; actin ACTG1; α-tubulin TUBA4; catechol o-methyltransferase COMT, which has been associated with a reduction in β-adrenergic sensitivity in a heart failure mouse model [36]; and eukaryotic initiation factor 4 subunit EIF4A2, which is a component of EIF4 pathway, a central pathway of protein translation. In addition, another subunit of EIF4, EIF4A3, was identified as significantly increased in HFpEF vs. HFrEF in a human patient plasma sample proteomic screening [37].

On the other hand, serum amyloid A-1 protein (SAA1), a circulatory acute-phase protein produced in response to inflammation, was detected as down-regulated in HFpEF myocardium samples. Biopsy samples had different degrees of blood contamination; therefore, the presence of circulatory proteins, such as SAA1, might be artificially differentially expressed between groups. The down-regulation of SAA1 was therefore devalued in our analysis.

### 3.2. Proteomic Signature of Myocardium Tissue from HFpEF Patients with Diabetes Mellitus

DM is one of the most prevalent comorbidities associated with HFpEF onset and aggravation. To assess the impact of diabetes on the proteomic profile of HFpEF myocardium, we analyzed and compared protein-expression profiles of HFpEF + DM samples vs. HFpEF samples of patients without DM diagnosis. Of the 1015 identified proteins, 78 presented significantly different expression levels (*p*-value ≤ 0.05) when comparing both sample groups (Supplementary File S5). 

**Figure 2:**
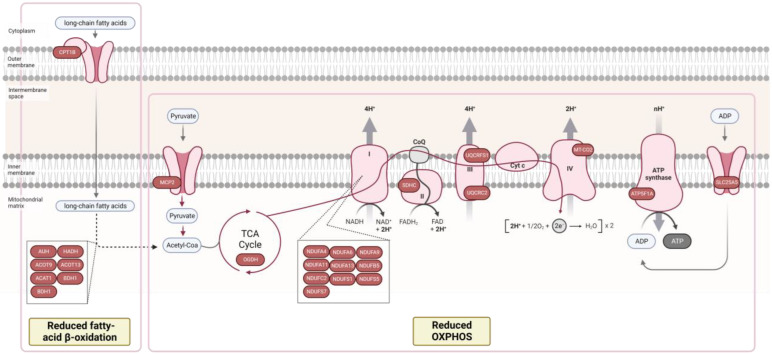
Proteomic analysis reveals decreased fatty-acid β oxidation and oxidative phosphorylation in myocardial tissue of patients with HFpEF + diabetes mellitus (DM). Proteins in the figure were identified as downregulated in HFpEF + DM group. Only proteins with a *p*-value ≤ 0.05 were included in the analysis. Figure adapted from “Electron Transport Chain”, by BioRender.com (2022). Retrieved from https://app.biorender.com/biorender-templates, accessed on 15 September 2022.

Besides increased systemic inflammation, insulin resistance in DM leads to higher levels of free fatty acid and excess glucose, which have been described as resulting in an overall reduction in oxidative phosphorylation and the generation of mitochondrial ATP, excessive ROS production, and alterations in mitochondrial fatty acid β oxidation [38,39,40]. Our proteomic analysis is in line with these reports (Figure 2). Namely, several members of all mitochondrial electron transport chain complexes were identified as down-regulated in HFpEF + DM samples: complex I (NADH dehydrogenase—NDUFA4, NDUFA6, NDUFA9, NDUFA11, NDUFA13, NDUFB5, NDUFC2, NDUFS1, NDUFS5, and NDUFS7), complex II (succinate dehydrogenase—SDHC), complex III (cytochrome reductase—UQCRFS1 and UQCRC2), complex IV (cytochrome c oxidase—MT-CO2), and complex V (ATP synthase—ATP5F1A). In addition, other mitochondrial proteins associated with maintaining mitochondria integrity (MICOS13, OPA1, AFG3L2, FIS1, and PHB2) and mitochondria carrier proteins SLC25A5, SLC25A11, SLC25A12, and MCP2 (which are responsible for the uptake of pyruvate) were also identified as down-regulated, indicating mitochondrial structure impairments and the functional dysregulation in tissues of patients with DM.

Mitochondrial 2-oxoglutarate dehydrogenase (OGDH), part of a multienzyme complex that catalyzes the oxidative decarboxylation of α-ketoglutarate to succinyl-coenzyme A, acting as a rate-limiting enzyme of the tricarboxylic acid cycle, was also identified as downregulated in HFpEF+DM samples (FC = 0.37), indicating the dysregulation of overall cellular metabolism in the myocardium of HFpEF+DM patients (Figure 2). The inhibition of this enzyme had been previously associated with DM in mice endothelial cells [41], and in the hearts of diabetic mice [42]. 

Fatty acids β oxidation also occurs in mitochondria and is a major energy source to sustain the energy requirements of the adult heart muscle [43,44]. In HF, PPARα transcriptional factor downregulation results in the decreased expression of several genes that regulate fatty acid uptake and oxidation, resulting in the overall inhibition of this pathway [40,45,46]. However, the increased levels of circulating fatty acids in diabetes tend to result in an opposite effect, with the up-regulation of fatty acid oxidation [39,40]. Therefore, several therapies applied in HF diabetic patients target circulating fatty acids and the downregulation of fatty acid oxidation, such as PPARα agonists, MCD inhibitors, and trimetazidine. In our results, eight key enzymes in this pathway were identified as down-regulated in HFpEF + DM samples, including CPT1B, an enzyme required for the transportation of long-chain fatty acids from the cytoplasm into the mitochondria, as well as other enzymes involved in fatty acids β oxidation (HAUH, ADH, ACAT1, ACOT9, ACOT13, DBT, and BDHT) (Figure 2), which could possibly be explained by the pharmacological therapy of this cohort of patients (unknown data).

Interestingly, several proteins involved in cardiac muscle contraction were also identified as downregulated, including myosins MYH2 and MYH7; titin (TTN); and cardiac actin ACTC1, CAPZA2, and MYOM3.

Two proteins identified as up-regulated in HFpEF + DM, DCD-2 (FC = 1.97) and GLRX (FC = 1.81) have been previously reported to have increased levels in the serum of diabetic patients [47,48].

We are aware that the study presents some limitations. Due to the inherent low availability of human heart ventricle biopsies, a small cohort of samples was analyzed, impacting the strength/statistical power of the findings. The groups analyzed were not perfectly matched in terms of clinical characteristics, age, cardiovascular comorbidities, the anatomical site of the myocardial sample collection, and contamination with blood. Therefore, we cannot exclude that other confounding factors and parameters besides HFpEF diagnosis and comorbidities (DM) may be hampering stronger results. More than the variability introduced by tissue-sample harvesting, the HFpEF broad range of phenotypical variations might hamper uncovering, whose proteins and signaling pathways are differentially expressed in these patients, leading to a lower statistical power of the analysis performed in this work. The co-occurrence of DM further increases the heterogeneity of the HFpEF population.

## 4. Conclusions

Overall, we have identified the increased expression of several proteins associated with protection against oxidative stress and the negative regulation of inflammation in HFpEF, which might constitute a reaction of the myocardium to the inflammatory environment and oxidative imbalance (both hallmarks of HFpEF cellular physiology). In addition, within HFpEF, the tissue samples of patients with DM presented protein signatures that correlated with oxidative phosphorylation, fatty acid β oxidation, mitochondrial function, and contractility impairments.

From these analyses, the deleterious impact that HFpEF and DM have on mitochondrial function and metabolism is clear: there is a need to develop drugs targeting the mitochondria. Despite the inherent disadvantage of human myocardial biopsies scarcity, leading to lower biological replicates and the small statistical power of studies using these types of samples, we strongly believe that investigating proteomic profiles and signaling pathways directly in human tissue samples brings us one step closer to the disease phenotype, providing new relevant insights into human HFpEF and DM biology at the myocardium cellular level. 

Machine and statistical-learning models applied to omics datasets are being increasingly explored to classify patient cohorts and predict pathologies and associated risks in a broad range of diseases, including HFpEF [49]. The data generated herein constitutes a valuable resource for additional analysis contributing to a better understanding of the disease’s underlying cellular mechanisms. We believe that the untargeted and thorough characterization of human biopsies is a key step for potentiating the further development of novel diagnosis strategies and clinical approaches for the targeted and precision treatment of HFpEF patients.

## Figures and Tables

**Table 1:** Clinical characteristics of patients. Categorical variables are presented by percentages (%), and continuous variables are given by median (Q1, Q3).

Parameters	HFpEF (n = 16)	Non-HFpEF (n = 11)	*p*-Value
Age, y	70 (67, 74)	58 (40, 68)	0.018
Male (%)	63	27	0.239
Diabetes mellitus (%)	67	27	0.047
Hypertension (%)	87	100	>0.999
Obesity (%)	40	43	>0.999
Anemia (%)	18	14	>0.999
Dyslipidemia (%)	91	86	>0.999

**Table 2:** List of significantly regulated proteins between HFpEF vs. non-HFpEF samples. Fold change (FC) values are depicted as red (FC ≤ 0.67) or green (FC ≥ 1.5, dark green if FC ≥ 1.5). Only proteins with a *p*-value ≤ 0.05 were considered for analysis.

ID	Name	FC	*p*-Value
EIF4A2	Eukaryotic initiation factor 4A-II	3.00	0.028
ACTG1	Actin, cytoplasmic 2	2.27	0.049
TUBA4A	Tubulin alpha-4A chain	2.10	0.035
NDUFA11	NADH dehydrogenase [ubiquinone] 1 alpha subcomplex subunit 11	2.06	0.049
PRXL2A	Peroxiredoxin-like 2A	2.00	0.038
AIFM1	Apoptosis-inducing factor 1, mitochondrial	1.84	0.045
IGKV3D-20	Immunoglobulin kappa variable 3D-20	1.78	0.048
PRKACA	cAMP-dependent protein kinase catalytic subunit alpha	1.77	0.048
LAMA4	Laminin subunit alpha-4	1.67	0.045
COMT	Catechol O-methyltransferase	1.46	0.029
EHD1	EH domain-containing protein 1	1.36	0.035
SAA1	Serum amyloid A-1 protein	0.42	0.046

## Data Availability

All proteomic raw data have been deposited in the ProteomeXchange Consortium (http://proteomecentral.proteomexchange.org, data submitted 12 May 2022) via the PRIDE partner repository with the dataset identifier PXD033876.

## Supplementary Materials

The following supporting information can be downloaded at https://www.mdpi.com/article/10.3390/biomedicines10112943/s1, File S1: Clinical characteristics of patients; File S2: Full list of quantified proteins by SWATH–MS analysis; File S3: Full list of normalized and batch-corrected quantified proteins; File S4: List of proteins identified in HFpEF vs. non HFpEF samples; File S5: List of proteins identified in HFpEF + diabetes melitus vs. HFpEF no diabetes melitus samples.
